# 3-Ethyl-4-phen­oxy-1-(2,2,2-trifluoro­eth­yl)-1*H*-pyrazol-5-ol

**DOI:** 10.1107/S1600536810025948

**Published:** 2010-07-07

**Authors:** Tara Shahani, Hoong-Kun Fun, R. Venkat Ragavan, V. Vijayakumar, S. Sarveswari

**Affiliations:** a X-ray Crystallography Unit, School of Physics, Universiti Sains Malaysia, 11800 USM, Penang, Malaysia; b Organic Chemistry Division, School of Advanced Sciences, VIT University, Vellore 632 014, India

## Abstract

The title compound, C_13_H_13_F_3_N_2_O_2_, crystallizes with two independent mol­ecules in the asymmetric unit, with different conformations of their ethyl side chains. The dihedral angles formed between the 1*H*-pyrazole and benzene rings in the two mol­ecules are 79.44 (6) and 77.81 (6)°. In the crystal, mol­ecules are linked by O⋯H—N hydrogen bonds into chains propagating along [001] and the packing is further stabilized by π–π inter­actions [centroid–centroid separations = 3.5409 (10) and 3.6335 (10) Å].

## Related literature

For the synthesis, see: Ragavan *et al.* (2009 ▶, 2010 ▶). For background on the biological activity of 3-ethyl-4-phen­oxy-1-(2,2,2-trifluoro­eth­yl)-1*H*-pyrazol-5-ol, see: Brogden (1986 ▶); Gursoy *et al.* (2000 ▶); Watanabe *et al.* (1984 ▶); Kawai *et al.* (1997 ▶); Wu *et al.* (2002 ▶). For related structures, see: Shahani *et al.* (2009 ▶, 2010*a* ▶,*b* ▶,*c* ▶,*d* ▶). For hydrogen-bond motifs, see: Bernstein *et al.* (1995 ▶). For bond-length data, see: Allen *et al.* (1987 ▶). For the stability of the temperature controller used for the data collection, see: Cosier & Glazer (1986 ▶). For related literature, see: Coersmeier *et al.* (1986 ▶).
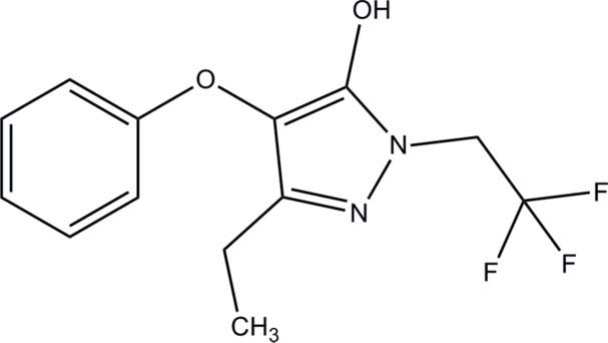


## Experimental

### 

#### Crystal data

C_13_H_13_F_3_N_2_O_2_
*M* *_r_* = 286.25
Monoclinic, 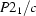

*a* = 9.3490 (18) Å
*b* = 14.712 (3) Å
*c* = 20.319 (4) Å
β = 113.889 (8)°
*V* = 2555.3 (9) Å^3^
*Z* = 8
Mo *K*α radiation
μ = 0.13 mm^−1^
*T* = 100 K
0.38 × 0.26 × 0.15 mm

#### Data collection

Bruker SMART APEXII CCD diffractometer
Absorption correction: multi-scan (*SADABS*; Bruker, 2009 ▶) *T*_min_ = 0.952, *T*_max_ = 0.981
35406 measured reflections
9653 independent reflections
7544 reflections with *I* > 2σ(*I*)
*R*_int_ = 0.034

#### Refinement

*R*[*F* ^2^ > 2σ(*F* ^2^)] = 0.046
*wR*(*F* ^2^) = 0.156
*S* = 1.07
9653 reflections
365 parameters
H-atom parameters constrained
Δρ_max_ = 0.68 e Å^−3^
Δρ_min_ = −0.55 e Å^−3^

### 

Data collection: *APEX2* (Bruker, 2009 ▶); cell refinement: *SAINT* (Bruker, 2009 ▶); data reduction: *SAINT*; program(s) used to solve structure: *SHELXTL* (Sheldrick, 2008 ▶); program(s) used to refine structure: *SHELXTL*; molecular graphics: *SHELXTL*; software used to prepare material for publication: *SHELXTL* and *PLATON* (Spek, 2009 ▶).

## Supplementary Material

Crystal structure: contains datablocks global, I. DOI: 10.1107/S1600536810025948/hb5540sup1.cif

Structure factors: contains datablocks I. DOI: 10.1107/S1600536810025948/hb5540Isup2.hkl

Additional supplementary materials:  crystallographic information; 3D view; checkCIF report

## Figures and Tables

**Table 1:** Selected torsion angles (°)

N1*A*—C8*A*—C12*A*—C13*A*	40.62 (16)
N1*B*—C8*B*—C12*B*—C13*B*	111.06 (16)

**Table 2:** Hydrogen-bond geometry (Å, °)

*D*—H⋯*A*	*D*—H	H⋯*A*	*D*⋯*A*	*D*—H⋯*A*
O2*A*—H1*OA*⋯N1*B*^i^	0.82	1.79	2.5996 (15)	169
O2*B*—H1*OB*⋯N1*A*^ii^	0.82	1.76	2.5781 (14)	177

Symmetry codes: (i) 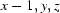; (ii) 
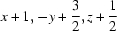
.

**Fig. 1.**
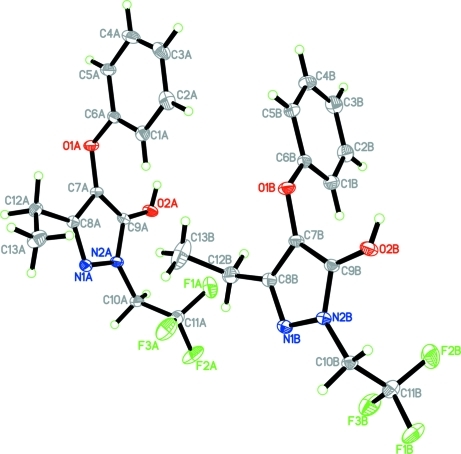
The molecular structure of (I), showing 45% probability displacement ellipsoids.

**Fig. 2.**
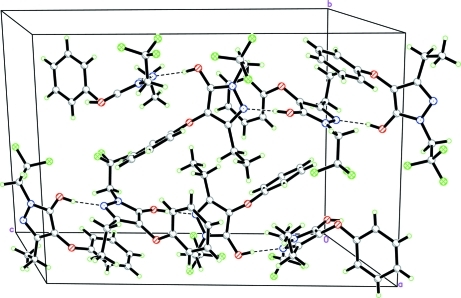
The crystal packing of (I), viewed approximately along a axis, showing one-dimensional chains along the [001] direction. Intermolecular hydrogen bonds are shown as dashed lines.

## supplementary crystallographic information

### Comment

Pyrazolone derivatives have a broad spectrum of biological activities being used as analgesic, antipyretic and anti-inflammatory therapeutical drugs (Brogden, 1986; Gursoy *et al.*, 2000). A class of new compounds with pyrazolone moiety was synthesized and reported for their antibacterial and antifungal activities (Ragavan *et al.*, 2009, 2010). A new pyrazolone derivative, edaravone (3-methyl-1-phenyl-2-pyrazoline-5-one), is being used as a drug in clinical practice for brain ischemia (Watanabe *et al.*, 1984; Kawai *et al.*, 1997) and the same has been found to be effective against myocardial ischemia (Wu *et al.*, 2002).

There are two independent molecules (A and B) in the asymmetric unit (Fig. 1). The maximum deviations in 1*H*-pyrazole rings (N1/N2/C7–C9) are 0.002 (1) and 0.003 (1) Å, respectively, for atom C7A of molecule A and atoms N2B and C8B of molecule B. The dihedral angles formed between the 1*H*-pyrazole rings and benzene rings in molecules A and B are 79.44 (6) and 77.81 (6)°, respectively. The bond lengths (Allen *et al.*,1987) and angles are within normal ranges and comparable to those closely related structures (Shahani *et al.*, 2009, 2010*a*,b,c,d).

In the crystal packing (Fig. 2), intermolecular O2A···H1OA—N1B, O2B···H1OB—N1A hydrogen bonds (Table 1) link the molecules into one-dimensional chains along [001] direction. The interesting feature of the crystal packing is provided by weak π–π interactions [Cg1···Cg4 = 3.5409 (10) Å, symmetry code, 1+*x*, 3/2-*y*, -1/2+*z*], [Cg2···Cg3 = 3.6335 (10) Å, symmetry code, -1 + *x*, *y*, *z*], *Cg*1 and *Cg*4 are the centroids of the benzene rings (C1A–C6A & C1B–C6B), *Cg*2 and *Cg*3 are the centroids of the 1*H*-pyrazole rings (N1A/N2A/C7A–C9A & N1B/N2B/C7B–C9B).

### Experimental

The title compound has been synthesized according to the available procedure in the literature (Ragavan *et al.*, 2009, 2010) and purified by column chromatography using ethyl acetate and methanol mixture (1:99). The obtained solid was recrystallized using absolute ethanol to yield colourless blocks of (I). Yield: 49%; *Mp*. 463–465 K.

### Refinement

H atoms were positioned geometrically [range of C–H = 0.93–0.97 Å, O–H = 0.82 Å] and refined using a riding model, with *U*_iso_(H) = 1.5 *U*_eq_(O) and 1.2 or 1.5 *U*_eq_(C). A rotating group model was used for the methyl groups.

### Crystal data

**Table:**

C_13_H_13_F_3_N_2_O_2_	*F*(000) = 1184
*M**_r_* = 286.25	*D*_x_ = 1.488 Mg m^−^^3^
Monoclinic, *P*2_1_/*c*	Mo *K*α radiation, λ = 0.71073 Å
Hall symbol: -P 2ybc	Cell parameters from 9950 reflections
*a* = 9.3490 (18) Å	θ = 2.5–33.1°
*b* = 14.712 (3) Å	µ = 0.13 mm^−^^1^
*c* = 20.319 (4) Å	*T* = 100 K
β = 113.889 (8)°	Block, colourless
*V* = 2555.3 (9) Å^3^	0.38 × 0.26 × 0.15 mm
*Z* = 8	

### Data collection

**Table:**

Bruker SMART APEXII CCD diffractometer	9653 independent reflections
Radiation source: fine-focus sealed tube	7544 reflections with *I* > 2σ(*I*)
graphite	*R*_int_ = 0.034
φ and ω scans	θ_max_ = 33.1°, θ_min_ = 2.2°
Absorption correction: multi-scan (*SADABS*; Bruker, 2009)	*h* = −14→13
*T*_min_ = 0.952, *T*_max_ = 0.981	*k* = −22→22
35406 measured reflections	*l* = −30→31

### Refinement

**Table:**

Refinement on *F*^2^	Primary atom site location: structure-invariant direct methods
Least-squares matrix: full	Secondary atom site location: difference Fourier map
*R*[*F*^2^ > 2σ(*F*^2^)] = 0.046	Hydrogen site location: inferred from neighbouring sites
*wR*(*F*^2^) = 0.156	H-atom parameters constrained
*S* = 1.07	*w* = 1/[σ^2^(*F*_o_^2^) + (0.0895*P*)^2^ + 0.5995*P*] where *P* = (*F*_o_^2^ + 2*F*_c_^2^)/3
9653 reflections	(Δ/σ)_max_ < 0.001
365 parameters	Δρ_max_ = 0.68 e Å^−^^3^
0 restraints	Δρ_min_ = −0.55 e Å^−^^3^

### Special details

**Table:**

Experimental. The crystal was placed in the cold stream of an Oxford Cyrosystems Cobra open-flow nitrogen cryostat (Cosier & Glazer, 1986) operating at 100.0 (1) K.
Geometry. All e.s.d.'s (except the e.s.d. in the dihedral angle between two l.s. planes) are estimated using the full covariance matrix. The cell e.s.d.'s are taken into account individually in the estimation of e.s.d.'s in distances, angles and torsion angles; correlations between e.s.d.'s in cell parameters are only used when they are defined by crystal symmetry. An approximate (isotropic) treatment of cell e.s.d.'s is used for estimating e.s.d.'s involving l.s. planes.
Refinement. Refinement of *F*^2^ against ALL reflections. The weighted *R*-factor *wR* and goodness of fit *S* are based on *F*^2^, conventional *R*-factors *R* are based on *F*, with *F* set to zero for negative *F*^2^. The threshold expression of *F*^2^ > σ(*F*^2^) is used only for calculating *R*-factors(gt) *etc*. and is not relevant to the choice of reflections for refinement. *R*-factors based on *F*^2^ are statistically about twice as large as those based on *F*, and *R*- factors based on ALL data will be even larger.

### Fractional atomic coordinates and isotropic or equivalent isotropic displacement parameters (Å^2^)

**Table:**

	*x*	*y*	*z*	*U*_iso_*/*U*_eq_	
F1A	0.31537 (13)	0.95145 (9)	0.55763 (5)	0.0481 (3)	
F2A	0.30166 (11)	1.01373 (6)	0.45960 (6)	0.0391 (2)	
F3A	0.39030 (10)	0.87810 (6)	0.48585 (6)	0.0382 (2)	
O1A	−0.02252 (9)	0.65761 (6)	0.57549 (4)	0.01824 (16)	
O2A	−0.02449 (11)	0.86757 (6)	0.54830 (5)	0.01993 (16)	
H1OA	−0.0368	0.8517	0.5843	0.030*	
N1A	0.14431 (11)	0.73591 (6)	0.45565 (5)	0.01659 (17)	
N2A	0.09635 (11)	0.81452 (6)	0.47642 (5)	0.01577 (17)	
C1A	0.24276 (14)	0.64218 (8)	0.66821 (6)	0.0199 (2)	
H1AA	0.2857	0.6722	0.6402	0.024*	
C2A	0.33808 (16)	0.61131 (9)	0.73676 (7)	0.0260 (2)	
H2AA	0.4452	0.6223	0.7551	0.031*	
C3A	0.27568 (18)	0.56449 (9)	0.77815 (7)	0.0292 (3)	
H3AA	0.3405	0.5438	0.8238	0.035*	
C4A	0.11638 (18)	0.54869 (9)	0.75105 (7)	0.0267 (3)	
H4AA	0.0744	0.5166	0.7785	0.032*	
C5A	0.01825 (15)	0.58036 (8)	0.68319 (6)	0.0212 (2)	
H5AA	−0.0890	0.5702	0.6654	0.025*	
C6A	0.08262 (13)	0.62747 (7)	0.64225 (6)	0.01685 (19)	
C7A	0.03786 (12)	0.70534 (7)	0.53426 (6)	0.01559 (18)	
C8A	0.10829 (12)	0.66915 (7)	0.49077 (6)	0.01611 (18)	
C9A	0.03091 (12)	0.79864 (7)	0.52395 (5)	0.01559 (18)	
C10A	0.11837 (13)	0.90055 (7)	0.44853 (6)	0.01736 (19)	
H10A	0.0446	0.9442	0.4525	0.021*	
H10B	0.0972	0.8940	0.3979	0.021*	
C11A	0.28240 (15)	0.93581 (9)	0.48839 (7)	0.0248 (2)	
C12A	0.13741 (14)	0.57127 (8)	0.48058 (6)	0.0201 (2)	
H12A	0.0530	0.5494	0.4372	0.024*	
H12B	0.1344	0.5368	0.5207	0.024*	
C13A	0.29193 (16)	0.55208 (9)	0.47496 (9)	0.0284 (3)	
H13A	0.3039	0.4877	0.4713	0.043*	
H13B	0.3765	0.5749	0.5171	0.043*	
H13C	0.2928	0.5816	0.4330	0.043*	
F1B	1.40543 (10)	0.97140 (6)	0.72206 (5)	0.0347 (2)	
F2B	1.29257 (11)	0.98298 (6)	0.79574 (5)	0.0353 (2)	
F3B	1.15899 (10)	0.99932 (6)	0.68199 (5)	0.0337 (2)	
O1B	0.86609 (10)	0.71693 (6)	0.80232 (5)	0.01864 (16)	
O2B	1.21261 (10)	0.76971 (6)	0.84486 (4)	0.01944 (16)	
H1OB	1.1880	0.7673	0.8792	0.029*	
N1B	0.96106 (11)	0.83472 (7)	0.67117 (5)	0.01796 (18)	
N2B	1.10254 (11)	0.82572 (7)	0.72879 (5)	0.01590 (17)	
C1B	0.75881 (14)	0.85277 (8)	0.83482 (6)	0.0203 (2)	
H1BA	0.8011	0.8909	0.8108	0.024*	
C2B	0.67177 (15)	0.88829 (9)	0.87052 (7)	0.0243 (2)	
H2BA	0.6560	0.9507	0.8703	0.029*	
C3B	0.60848 (15)	0.83187 (10)	0.90634 (7)	0.0265 (2)	
H3BA	0.5508	0.8563	0.9301	0.032*	
C4B	0.63163 (15)	0.73870 (10)	0.90657 (7)	0.0250 (2)	
H4BA	0.5890	0.7006	0.9304	0.030*	
C5B	0.71822 (14)	0.70211 (8)	0.87132 (6)	0.0198 (2)	
H5BA	0.7339	0.6397	0.8715	0.024*	
C6B	0.78125 (12)	0.75984 (8)	0.83571 (6)	0.01636 (19)	
C7B	0.92916 (13)	0.76861 (7)	0.76383 (6)	0.01631 (19)	
C8B	0.85532 (13)	0.80022 (8)	0.69294 (6)	0.0185 (2)	
C9B	1.08731 (13)	0.78561 (7)	0.78566 (6)	0.01529 (18)	
C10B	1.24605 (13)	0.85110 (7)	0.72379 (6)	0.01700 (19)	
H10C	1.2429	0.8313	0.6777	0.020*	
H10D	1.3323	0.8200	0.7612	0.020*	
C11B	1.27517 (14)	0.95189 (8)	0.73120 (7)	0.0229 (2)	
C12B	0.68516 (15)	0.80124 (9)	0.64426 (7)	0.0249 (2)	
H12C	0.6700	0.8390	0.6028	0.030*	
H12D	0.6277	0.8288	0.6696	0.030*	
C13B	0.6182 (2)	0.70828 (12)	0.61840 (12)	0.0492 (5)	
H13D	0.5089	0.7137	0.5879	0.074*	
H13E	0.6311	0.6707	0.6590	0.074*	
H13F	0.6720	0.6813	0.5918	0.074*	

### Atomic displacement parameters (Å^2^)

**Table:**

	*U*^11^	*U*^22^	*U*^33^	*U*^12^	*U*^13^	*U*^23^
F1A	0.0471 (6)	0.0628 (7)	0.0289 (5)	−0.0242 (5)	0.0099 (4)	−0.0171 (5)
F2A	0.0398 (5)	0.0210 (4)	0.0647 (7)	−0.0077 (3)	0.0296 (5)	0.0043 (4)
F3A	0.0216 (4)	0.0297 (4)	0.0623 (6)	0.0010 (3)	0.0160 (4)	0.0024 (4)
O1A	0.0181 (3)	0.0218 (4)	0.0161 (3)	−0.0019 (3)	0.0082 (3)	0.0050 (3)
O2A	0.0276 (4)	0.0192 (4)	0.0184 (4)	0.0039 (3)	0.0149 (3)	0.0020 (3)
N1A	0.0206 (4)	0.0159 (4)	0.0158 (4)	−0.0008 (3)	0.0100 (3)	−0.0014 (3)
N2A	0.0199 (4)	0.0153 (4)	0.0150 (4)	−0.0001 (3)	0.0101 (3)	0.0011 (3)
C1A	0.0217 (5)	0.0171 (5)	0.0202 (5)	0.0014 (4)	0.0077 (4)	−0.0001 (4)
C2A	0.0282 (6)	0.0220 (5)	0.0226 (5)	0.0066 (4)	0.0051 (4)	−0.0006 (4)
C3A	0.0440 (7)	0.0223 (5)	0.0177 (5)	0.0108 (5)	0.0089 (5)	0.0034 (4)
C4A	0.0456 (7)	0.0193 (5)	0.0206 (5)	0.0066 (5)	0.0188 (5)	0.0048 (4)
C5A	0.0309 (6)	0.0169 (5)	0.0200 (5)	0.0010 (4)	0.0148 (4)	0.0026 (4)
C6A	0.0230 (5)	0.0143 (4)	0.0141 (4)	0.0012 (4)	0.0084 (4)	0.0006 (3)
C7A	0.0175 (4)	0.0167 (4)	0.0142 (4)	−0.0018 (3)	0.0081 (3)	0.0018 (3)
C8A	0.0170 (4)	0.0170 (4)	0.0152 (4)	−0.0015 (3)	0.0074 (3)	−0.0006 (3)
C9A	0.0175 (4)	0.0181 (5)	0.0128 (4)	0.0005 (3)	0.0077 (3)	0.0009 (3)
C10A	0.0202 (5)	0.0166 (4)	0.0173 (4)	0.0000 (4)	0.0098 (4)	0.0029 (3)
C11A	0.0253 (5)	0.0220 (5)	0.0289 (6)	−0.0051 (4)	0.0127 (5)	−0.0011 (4)
C12A	0.0226 (5)	0.0167 (5)	0.0232 (5)	−0.0024 (4)	0.0116 (4)	−0.0012 (4)
C13A	0.0291 (6)	0.0194 (5)	0.0423 (7)	0.0025 (4)	0.0202 (6)	0.0021 (5)
F1B	0.0263 (4)	0.0322 (4)	0.0462 (5)	−0.0103 (3)	0.0154 (4)	0.0059 (4)
F2B	0.0439 (5)	0.0260 (4)	0.0346 (5)	−0.0037 (4)	0.0145 (4)	−0.0125 (3)
F3B	0.0298 (4)	0.0219 (4)	0.0432 (5)	0.0013 (3)	0.0084 (4)	0.0115 (3)
O1B	0.0237 (4)	0.0165 (3)	0.0220 (4)	−0.0016 (3)	0.0158 (3)	0.0007 (3)
O2B	0.0189 (4)	0.0267 (4)	0.0143 (3)	0.0009 (3)	0.0083 (3)	0.0028 (3)
N1B	0.0187 (4)	0.0217 (4)	0.0146 (4)	−0.0016 (3)	0.0078 (3)	0.0006 (3)
N2B	0.0175 (4)	0.0183 (4)	0.0141 (4)	−0.0015 (3)	0.0086 (3)	0.0000 (3)
C1B	0.0225 (5)	0.0193 (5)	0.0210 (5)	−0.0012 (4)	0.0107 (4)	0.0003 (4)
C2B	0.0240 (5)	0.0245 (6)	0.0252 (5)	0.0026 (4)	0.0108 (4)	−0.0031 (4)
C3B	0.0221 (5)	0.0351 (7)	0.0264 (6)	0.0010 (5)	0.0141 (5)	−0.0052 (5)
C4B	0.0229 (5)	0.0321 (6)	0.0257 (6)	−0.0047 (5)	0.0158 (5)	−0.0004 (5)
C5B	0.0198 (5)	0.0214 (5)	0.0208 (5)	−0.0032 (4)	0.0109 (4)	0.0007 (4)
C6B	0.0155 (4)	0.0196 (5)	0.0154 (4)	−0.0018 (3)	0.0078 (3)	−0.0009 (3)
C7B	0.0187 (4)	0.0171 (4)	0.0163 (4)	−0.0014 (4)	0.0104 (4)	0.0004 (3)
C8B	0.0191 (5)	0.0207 (5)	0.0169 (4)	−0.0022 (4)	0.0085 (4)	0.0001 (4)
C9B	0.0191 (4)	0.0153 (4)	0.0146 (4)	−0.0001 (3)	0.0101 (4)	−0.0006 (3)
C10B	0.0187 (4)	0.0171 (4)	0.0189 (4)	−0.0010 (4)	0.0114 (4)	−0.0004 (4)
C11B	0.0225 (5)	0.0189 (5)	0.0269 (6)	−0.0023 (4)	0.0098 (4)	0.0009 (4)
C12B	0.0214 (5)	0.0276 (6)	0.0238 (5)	−0.0013 (4)	0.0072 (4)	0.0018 (4)
C13B	0.0315 (8)	0.0303 (8)	0.0669 (12)	−0.0082 (6)	0.0004 (8)	−0.0036 (8)

### Geometric parameters (Å, °)

**Table:**

F1A—C11A	1.3326 (17)	F1B—C11B	1.3354 (15)
F2A—C11A	1.3319 (16)	F2B—C11B	1.3351 (16)
F3A—C11A	1.3354 (16)	F3B—C11B	1.3367 (15)
O1A—C7A	1.3773 (13)	O1B—C7B	1.3822 (13)
O1A—C6A	1.3866 (13)	O1B—C6B	1.3871 (13)
O2A—C9A	1.3224 (13)	O2B—C9B	1.3162 (13)
O2A—H1OA	0.8200	O2B—H1OB	0.8200
N1A—C8A	1.3349 (14)	N1B—C8B	1.3360 (14)
N1A—N2A	1.3675 (13)	N1B—N2B	1.3725 (13)
N2A—C9A	1.3566 (13)	N2B—C9B	1.3554 (13)
N2A—C10A	1.4352 (14)	N2B—C10B	1.4354 (14)
C1A—C6A	1.3881 (16)	C1B—C6B	1.3822 (16)
C1A—C2A	1.3910 (17)	C1B—C2B	1.3928 (17)
C1A—H1AA	0.9300	C1B—H1BA	0.9300
C2A—C3A	1.386 (2)	C2B—C3B	1.3848 (19)
C2A—H2AA	0.9300	C2B—H2BA	0.9300
C3A—C4A	1.382 (2)	C3B—C4B	1.387 (2)
C3A—H3AA	0.9300	C3B—H3BA	0.9300
C4A—C5A	1.3911 (17)	C4B—C5B	1.3884 (17)
C4A—H4AA	0.9300	C4B—H4BA	0.9300
C5A—C6A	1.3929 (15)	C5B—C6B	1.3919 (15)
C5A—H5AA	0.9300	C5B—H5BA	0.9300
C7A—C9A	1.3861 (15)	C7B—C9B	1.3836 (15)
C7A—C8A	1.4032 (15)	C7B—C8B	1.4009 (15)
C8A—C12A	1.4954 (16)	C8B—C12B	1.4949 (17)
C10A—C11A	1.5070 (17)	C10B—C11B	1.5042 (16)
C10A—H10A	0.9700	C10B—H10C	0.9700
C10A—H10B	0.9700	C10B—H10D	0.9700
C12A—C13A	1.5209 (18)	C12B—C13B	1.507 (2)
C12A—H12A	0.9700	C12B—H12C	0.9700
C12A—H12B	0.9700	C12B—H12D	0.9700
C13A—H13A	0.9600	C13B—H13D	0.9600
C13A—H13B	0.9600	C13B—H13E	0.9600
C13A—H13C	0.9600	C13B—H13F	0.9600
			
C7A—O1A—C6A	117.08 (9)	C7B—O1B—C6B	119.07 (9)
C9A—O2A—H1OA	109.5	C9B—O2B—H1OB	109.5
C8A—N1A—N2A	105.79 (9)	C8B—N1B—N2B	105.52 (9)
C9A—N2A—N1A	111.89 (9)	C9B—N2B—N1B	111.82 (9)
C9A—N2A—C10A	127.67 (9)	C9B—N2B—C10B	126.69 (9)
N1A—N2A—C10A	120.44 (9)	N1B—N2B—C10B	121.34 (9)
C6A—C1A—C2A	119.00 (11)	C6B—C1B—C2B	118.81 (11)
C6A—C1A—H1AA	120.5	C6B—C1B—H1BA	120.6
C2A—C1A—H1AA	120.5	C2B—C1B—H1BA	120.6
C3A—C2A—C1A	120.90 (13)	C3B—C2B—C1B	120.87 (12)
C3A—C2A—H2AA	119.5	C3B—C2B—H2BA	119.6
C1A—C2A—H2AA	119.5	C1B—C2B—H2BA	119.6
C4A—C3A—C2A	119.49 (12)	C2B—C3B—C4B	119.64 (11)
C4A—C3A—H3AA	120.3	C2B—C3B—H3BA	120.2
C2A—C3A—H3AA	120.3	C4B—C3B—H3BA	120.2
C3A—C4A—C5A	120.68 (12)	C3B—C4B—C5B	120.27 (11)
C3A—C4A—H4AA	119.7	C3B—C4B—H4BA	119.9
C5A—C4A—H4AA	119.7	C5B—C4B—H4BA	119.9
C4A—C5A—C6A	119.18 (12)	C4B—C5B—C6B	119.31 (11)
C4A—C5A—H5AA	120.4	C4B—C5B—H5BA	120.3
C6A—C5A—H5AA	120.4	C6B—C5B—H5BA	120.3
O1A—C6A—C1A	123.38 (10)	C1B—C6B—O1B	123.89 (10)
O1A—C6A—C5A	115.90 (10)	C1B—C6B—C5B	121.09 (10)
C1A—C6A—C5A	120.72 (11)	O1B—C6B—C5B	115.02 (10)
O1A—C7A—C9A	126.23 (10)	O1B—C7B—C9B	124.29 (10)
O1A—C7A—C8A	127.02 (10)	O1B—C7B—C8B	128.23 (10)
C9A—C7A—C8A	106.62 (9)	C9B—C7B—C8B	106.69 (9)
N1A—C8A—C7A	109.98 (10)	N1B—C8B—C7B	110.12 (10)
N1A—C8A—C12A	122.34 (10)	N1B—C8B—C12B	120.60 (10)
C7A—C8A—C12A	127.65 (10)	C7B—C8B—C12B	129.26 (10)
O2A—C9A—N2A	119.48 (10)	O2B—C9B—N2B	119.65 (10)
O2A—C9A—C7A	134.79 (10)	O2B—C9B—C7B	134.49 (10)
N2A—C9A—C7A	105.72 (9)	N2B—C9B—C7B	105.84 (9)
N2A—C10A—C11A	111.64 (10)	N2B—C10B—C11B	112.69 (9)
N2A—C10A—H10A	109.3	N2B—C10B—H10C	109.1
C11A—C10A—H10A	109.3	C11B—C10B—H10C	109.1
N2A—C10A—H10B	109.3	N2B—C10B—H10D	109.1
C11A—C10A—H10B	109.3	C11B—C10B—H10D	109.1
H10A—C10A—H10B	108.0	H10C—C10B—H10D	107.8
F2A—C11A—F1A	107.48 (11)	F2B—C11B—F1B	107.93 (11)
F2A—C11A—F3A	106.96 (11)	F2B—C11B—F3B	107.15 (11)
F1A—C11A—F3A	107.17 (12)	F1B—C11B—F3B	107.11 (10)
F2A—C11A—C10A	110.45 (11)	F2B—C11B—C10B	112.41 (10)
F1A—C11A—C10A	112.14 (11)	F1B—C11B—C10B	109.64 (10)
F3A—C11A—C10A	112.37 (11)	F3B—C11B—C10B	112.36 (10)
C8A—C12A—C13A	114.89 (10)	C8B—C12B—C13B	113.70 (12)
C8A—C12A—H12A	108.5	C8B—C12B—H12C	108.8
C13A—C12A—H12A	108.5	C13B—C12B—H12C	108.8
C8A—C12A—H12B	108.5	C8B—C12B—H12D	108.8
C13A—C12A—H12B	108.5	C13B—C12B—H12D	108.8
H12A—C12A—H12B	107.5	H12C—C12B—H12D	107.7
C12A—C13A—H13A	109.5	C12B—C13B—H13D	109.5
C12A—C13A—H13B	109.5	C12B—C13B—H13E	109.5
H13A—C13A—H13B	109.5	H13D—C13B—H13E	109.5
C12A—C13A—H13C	109.5	C12B—C13B—H13F	109.5
H13A—C13A—H13C	109.5	H13D—C13B—H13F	109.5
H13B—C13A—H13C	109.5	H13E—C13B—H13F	109.5
			
C8A—N1A—N2A—C9A	0.04 (12)	C8B—N1B—N2B—C9B	0.58 (12)
C8A—N1A—N2A—C10A	179.76 (10)	C8B—N1B—N2B—C10B	176.38 (10)
C6A—C1A—C2A—C3A	1.65 (18)	C6B—C1B—C2B—C3B	−0.03 (18)
C1A—C2A—C3A—C4A	−0.35 (19)	C1B—C2B—C3B—C4B	−0.16 (19)
C2A—C3A—C4A—C5A	−0.87 (19)	C2B—C3B—C4B—C5B	0.2 (2)
C3A—C4A—C5A—C6A	0.75 (18)	C3B—C4B—C5B—C6B	−0.10 (19)
C7A—O1A—C6A—C1A	−0.79 (15)	C2B—C1B—C6B—O1B	−179.77 (11)
C7A—O1A—C6A—C5A	179.25 (10)	C2B—C1B—C6B—C5B	0.16 (17)
C2A—C1A—C6A—O1A	178.28 (10)	C7B—O1B—C6B—C1B	−1.95 (16)
C2A—C1A—C6A—C5A	−1.76 (17)	C7B—O1B—C6B—C5B	178.11 (10)
C4A—C5A—C6A—O1A	−179.46 (10)	C4B—C5B—C6B—C1B	−0.10 (17)
C4A—C5A—C6A—C1A	0.58 (17)	C4B—C5B—C6B—O1B	179.84 (10)
C6A—O1A—C7A—C9A	−103.42 (13)	C6B—O1B—C7B—C9B	109.05 (12)
C6A—O1A—C7A—C8A	81.34 (14)	C6B—O1B—C7B—C8B	−82.47 (14)
N2A—N1A—C8A—C7A	−0.27 (12)	N2B—N1B—C8B—C7B	−0.54 (13)
N2A—N1A—C8A—C12A	177.87 (10)	N2B—N1B—C8B—C12B	178.04 (10)
O1A—C7A—C8A—N1A	176.39 (10)	O1B—C7B—C8B—N1B	−169.75 (10)
C9A—C7A—C8A—N1A	0.39 (12)	C9B—C7B—C8B—N1B	0.33 (13)
O1A—C7A—C8A—C12A	−1.62 (18)	O1B—C7B—C8B—C12B	11.8 (2)
C9A—C7A—C8A—C12A	−177.61 (11)	C9B—C7B—C8B—C12B	−178.10 (12)
N1A—N2A—C9A—O2A	−178.67 (9)	N1B—N2B—C9B—O2B	178.28 (9)
C10A—N2A—C9A—O2A	1.64 (17)	C10B—N2B—C9B—O2B	2.75 (16)
N1A—N2A—C9A—C7A	0.20 (12)	N1B—N2B—C9B—C7B	−0.37 (12)
C10A—N2A—C9A—C7A	−179.49 (10)	C10B—N2B—C9B—C7B	−175.90 (10)
O1A—C7A—C9A—O2A	2.2 (2)	O1B—C7B—C9B—O2B	−7.8 (2)
C8A—C7A—C9A—O2A	178.26 (12)	C8B—C7B—C9B—O2B	−178.33 (12)
O1A—C7A—C9A—N2A	−176.39 (10)	O1B—C7B—C9B—N2B	170.60 (10)
C8A—C7A—C9A—N2A	−0.35 (12)	C8B—C7B—C9B—N2B	0.03 (12)
C9A—N2A—C10A—C11A	98.24 (13)	C9B—N2B—C10B—C11B	−104.92 (13)
N1A—N2A—C10A—C11A	−81.44 (12)	N1B—N2B—C10B—C11B	79.95 (13)
N2A—C10A—C11A—F2A	177.43 (10)	N2B—C10B—C11B—F2B	63.40 (13)
N2A—C10A—C11A—F1A	−62.72 (14)	N2B—C10B—C11B—F1B	−176.55 (10)
N2A—C10A—C11A—F3A	58.09 (14)	N2B—C10B—C11B—F3B	−57.56 (14)
N1A—C8A—C12A—C13A	40.62 (16)	N1B—C8B—C12B—C13B	111.06 (16)
C7A—C8A—C12A—C13A	−141.59 (12)	C7B—C8B—C12B—C13B	−70.65 (19)

### Hydrogen-bond geometry (Å, °)

**Table:**

*D*—H···*A*	*D*—H	H···*A*	*D*···*A*	*D*—H···*A*
O2A—H1OA···N1B^i^	0.82	1.79	2.5996 (15)	169
O2B—H1OB···N1A^ii^	0.82	1.76	2.5781 (14)	177

Symmetry codes: (i) *x*−1, *y*, *z*; (ii) *x*+1, −*y*+3/2, *z*+1/2.
